# Titanium dioxide modified with silver by two methods for bactericidal applications

**DOI:** 10.1016/j.heliyon.2019.e01608

**Published:** 2019-05-14

**Authors:** G. Durango-Giraldo, A. Cardona, Juan Felipe Zapata, Juan Felipe Santa, R. Buitrago-Sierra

**Affiliations:** a Materiales Avanzados y Energía – MATyER Research Group, Facultad de Ingeniería, Instituto Tecnológico Metropolitano-ITM, Medellín, Colombia; b Universidad Nacional de Colombia, Medellín, Colombia

**Keywords:** Materials science, Materials chemistry, Nanotechnology

## Abstract

“Titanium dioxide (TiO_2_) is a semiconductor material that exhibits antibacterial activity due to its photocatalytic properties under ultraviolet light. On the other hand, silver also exhibits strong antibacterial activity towards a wide range of microorganisms and TiO_2_ with silver addition exhibits more efficient photocatalytic properties than unmodified TiO_2_. In this work, TiO_2_ nanoparticles were synthesized by the hydrothermal method and modified with silver by two different methods: wet impregnation (*Ex situ*) and *In situ* incorporation. The antimicrobial activity of TiO_2_ nanoparticles synthesized and modified by both methods was evaluated against *Escherichia coli* and *Staphylococcus aureus*. The results showed that TiO_2_ nanoparticles have anatase phase. Also, spherical morphology with a mean particle size around 10.6 nm was obtained. The presence of silver in the modified TiO_2_ nanoparticles was confirmed by EDS and XPS. TiO_2_ particles modified by the *Ex situ* method, showed a better bactericidal activity compared to the particles modified by *In situ* incorporation method and TiO_2_ unmodified nanoparticles. This study demonstrated that both methods used to modify the titanium dioxide nanoparticles are effective as bactericidal materials and better results were found for the *Ex situ* method.”

## 1 Introduction

Titanium dioxide (TiO_2_) is a semiconductor material with three crystalline structures: Anatase, Rutile and Brookite. Anatase is the most popular TiO_2_ crystalline form, and it is commonly used in photocatalyst application due to its band gap energy of 3.2 eV [1]. TiO_2_ could be used in many different applications as white pigment [2], microbatteries [3, 4], acetone oxidation [5], air purification [6], anticorrosive coatings [7], UV absorber in cosmetic products [8] and for antibacterial materials [9, 10]. TiO_2_ particles are extensively known for their bactericidal effect when activated by UV light. When TiO_2_ is irradiated with energy greater than its band gap energy, an electron is excited from the valence band to the conduction band. As a result, electron-hole pairs are formed and react with water or oxygen molecules forming various reactive oxygen species (ROS) that are strong oxidants [11]. The ROS can cause oxidative damage to cell membranes and kill microorganisms [11]. However, the widespread technological use of TiO_2_ in photocatalysis is to some extent limited by its wide band gap of 3.2 eV, requiring UV light irradiation for photocatalytic activation. Since UV light accounts for only a small fraction (5%) of solar energy compared to visible light (∼ 50%) their uses in everyday applications are limited. The shift in the optical response of TiO_2_ from the UV to the visible light range will have a positive effect on its practical applications.

Different efforts have been made to increase applicability and efficiency of TiO_2_. Some of these works were based on TiO_2_ particles modification with metals such as silver [12], zinc [13] and cooper [14]. Previous studies reported that silver addition enhances the photocatalytic efficiency of titanium dioxide [14]. Silver shows a broad spectrum of antibacterial activity being active against gram-negative and gram-positive bacteria. The mechanism of action of Ag can be explained by three different mechanisms, the first one is caused by the release of toxic metal ions inhibiting the production of adenosine triphosphate (ATP) and deoxyribonucleic acid replication (fundamentals factors for the cellular survival). The second one is the generation of ROS that generates oxidative stress and cellular death, and the third one is damage to the cell membrane due to direct contact with nanoparticles [15]. Silver particles do not require UV light for inhibition of microorganisms, but in large quantities can be toxic. In the attempt of take advantage of both materials properties, numerous methods have been reported in the literature reports for the modification of TiO_2_ nanoparticles with Ag [16]. From this the most used methods are wet impregnation (or *Ex situ* method) and *In situ* or during synthesis. The wet impregnation method consists in the addition of a silver precursor to a solution of TiO_2_ nanoparticles under constant stirring, to achieve a good dispersion of silver precursor in the nanoparticles [17]. In the *In situ* method, the silver precursor is added during the synthesis of TiO_2_ nanoparticles. However, there are few reports in the literature relating the modification of titanium dioxide with silver by *Ex situ* and *In situ* method. In addition, the effect of the modification on photocatalytic activity and bactericide properties is also a topic of interest to obtain new materials with improved characteristics.

To the best of the authors knowledge and belief, the influence of the modification method of TiO_2_ on its bactericidal properties has not been studied. Accordingly, the aim of this work was to evaluate the effect of silver modification of TiO_2_ nanoparticles (using two different methods) on the bactericidal properties. The results obtained using modified TiO_2_ nanoparticles with 1% wt. of silver by wet impregnation method (Ag/TiO_2_-Ex) and *In situ* method (Ag/TiO_2_-In) are reported. The size, shape, crystalline structure and chemical composition of TiO_2_ nanoparticles synthesized by hydrothermal method and modified with silver were studied. Furthermore, the antibacterial activity of the TiO_2_ and Ag/TiO_2_ nanoparticles were investigated against Gram-positive *Staphylococcus aureus* (*S. aureus*), and Gram-negative *Escherichia coli* (*E. coli*). The contribution of this work is related to the comparison of two different synthesis methods and the evaluation of their effect on bactericidal applications.

## 2 Materials and methods

### 2.1 Materials

All the chemicals used were of reagent-grade and they were used without any further purification. Materials used for synthesis of pure TiO_2_ and Ag/TiO_2_ were titanium isopropoxide (Ti[OCH(CH_3_)_2_]_4_, 97% Sigma-Aldrich) and Silver nitrate (AgNO_3_, 99.9% Sigma-Aldrich) and ethanol (99.5% J.T Baker). All solutions were prepared using deionized water.

### 2.2 Synthesis of TiO_2_ and TiO_2_ modified with Ag (Ag/TiO_2_)

#### 2.2.1 Synthesis of TiO_2_ nanoparticles

TiO_2_ nanoparticles (NPs) were synthesized by hydrothermal method following the procedure reported by Kartini et al. [18]. In this method, titanium isopropoxide (TTIP) was used as precursor, ethanol as solvent and deionized water for the hydrolysis and condensation reaction. 10 mL of TTIP were added into 13.3 mL ethanol under constant stirring and then 16.6 mL of deionized water were added dropwise in the TTIP-ethanol under constant stirring. This mixture was stirred for 2 h and transferred to a Teflon lined autoclave and heated at 80 °C during 4 h in an oven furnace (Binder, KB 105). Finally, calcination at 400 °C for 4 h was done in a tube furnace (Nabertherm, P 330) to favor the formation of particles in the crystalline phase anatase and remove the waste products.

#### 2.2.2 TiO_2_ nanoparticles silver addition-Wet impregnation (*Ex situ*)

TiO_2_ particles with 1% wt. silver content were prepared by the wet impregnation method. To this, a solution of silver nitrate 1.9 mM in ethanol was prepared and 1 g of TiO_2_ nanoparticles (previously synthesized) were add to the solution. The resulting solution was constantly stirred for 6 h at room temperature and aged for 24 h. Finally, the solution was dried in an oven furnace at 80 °C, overnight and calcined at 450 °C for 5 h. The initial heating rate was maintained at 5 °C/min. The particles obtained were labeled as Ag/TiO_2_-Ex.

#### 2.2.3 TiO_2_ nanoparticles silver addition *In situ* method

Ag modified TiO_2_ particles with 1% wt of silver were also prepared by *In situ* method. Briefly, for the addition to silver at the synthesis, 0.032 g of silver nitrate was mixed with 50 mL of the deionized water. Subsequently, the mixture was added dropwise to the titanium precursor (TTIP) to control the hydrolysis process. After that, the solution was stirred during 6 hours. Finally, calcination at 450 °C for 4 h was done in an oven furnace. The particles obtained were labeled as Ag/TiO_2_-In

### 2.3 Antimicrobial test

Broth macrodilution was used to assess the antimicrobial activity of unmodified and modified TiO_2_ using the *Ex situ* and *In situ* methods. For the antimicrobial test, *Escherichia coli* (ATCC 25922) and *Staphylococcus aureus* (ATCC 43300) were selected as gram-negative and gram-positive bacteria models, respectively. For every experiment, bacteria stocks were kept at -20 °C and after that they were grown in BHI (Brain Heart Infusion) for 24 hours in an incubator at 37 °C and 80% of relative humidity. Then, the inoculum was prepared and adjusted until its absorbance was in the range reported for the McFarland's standard No. 0.5 (1–2 × 10^8^ CFU/ml; OD at 625 nm: 0.08–0.13) [19]. Accordingly, an absorbance of 0.09 was set for the inoculums. For the test of bactericidal activity, serial dilutions of TiO_2_, Ag/TiO_2_-Ex and Ag/TiO_2_-In from 800 μg/mL to 100 μg/mL were prepared and after that, 10 μL of the inoculum were placed into 2 mL tubes containing 1.5 mL of each serial dilution. As control, 10 μL of bacteria inoculum were placed in BHI or BHI supplemented with tetracycline. Subsequently, macrodilutions, controls, and BHI blanks containing the material concentrations tested, were incubated at 37 °C and 80% of relative humidity for 24 h under constant agitation (30 rpm). The bacteria concentrations were determined by measuring optical densities (OD) at 625 nm with a spectrophotometer (8453A, Agilent). macrodilutions and blanks were diluted and then measured. Experiments were performed by triplicate. Bacterial viability was calculated as follows:

Bacterial viability = *OD sample*/*OD control*

Where *OD sample* is the absorbance of each tested with the material dilutions and *OD control* is the absorbance of the sample untreated. Statistical analyses were performed in R v. 3.5.0 free software. For viability comparisons, linear models were initially implemented and then tested for model assumptions. One-way ANOVA was used if a model met assumptions, otherwise, Kruskal-Wallis tests were implemented. Parametric or non-parametric pairwise comparisons were conducted with the FDR correction method. *p*-value less than 0.05 was considered statistically significant.

## 3 Analysis

Phase identification and lattice structure of the synthesized NPs were characterized using an X-ray diffractometer (PANalytical Empyrean Series II), operated with Cu Kα radiation (λ = 1,540 Å) with an X-ray source generated at 45 keV and 40 mA. The shape of the particles was determined using a scanning electron microscope JEOL 7100F (FE-SEM) with a voltage of 15 kV and a working distance of 6–10 mm. Morphology and particles size were determined using a transmission electron microscope (TEM) FEI Tecnai F20 Super-Twin and the measurement by Image J free software. To this more than 300 particles by sample were measured. The functional groups in the sample were analyzed using a spectrophotometer IRTracer-100 (FTIR) with wavelengths between 400-4000 cm^−1^, by the diffuse reflectance infrared Fourier transform (DRIFT) method. Nanoparticles chemical composition, was obtained by Energy Dispersive Spectrometry (EDS) using a X-MAXN, OXFORD attached to a scanning electron microscope. Surface atomic composition and chemical state of the TiO_2_, Ag/TiO_2_-In and Ag/TiO_2_-Ex were characterized using an X-ray photoelectron spectroscopy (XPS, SPECS) with a PHOIBOS 150 1D-DLD analyzer and monochromatic Al Kα radiation (1487 eV) operated at 13 kV and 100W.

## 4 Results and discussion

### 4.1 Physical-chemical characterization

**Fig. 1:**
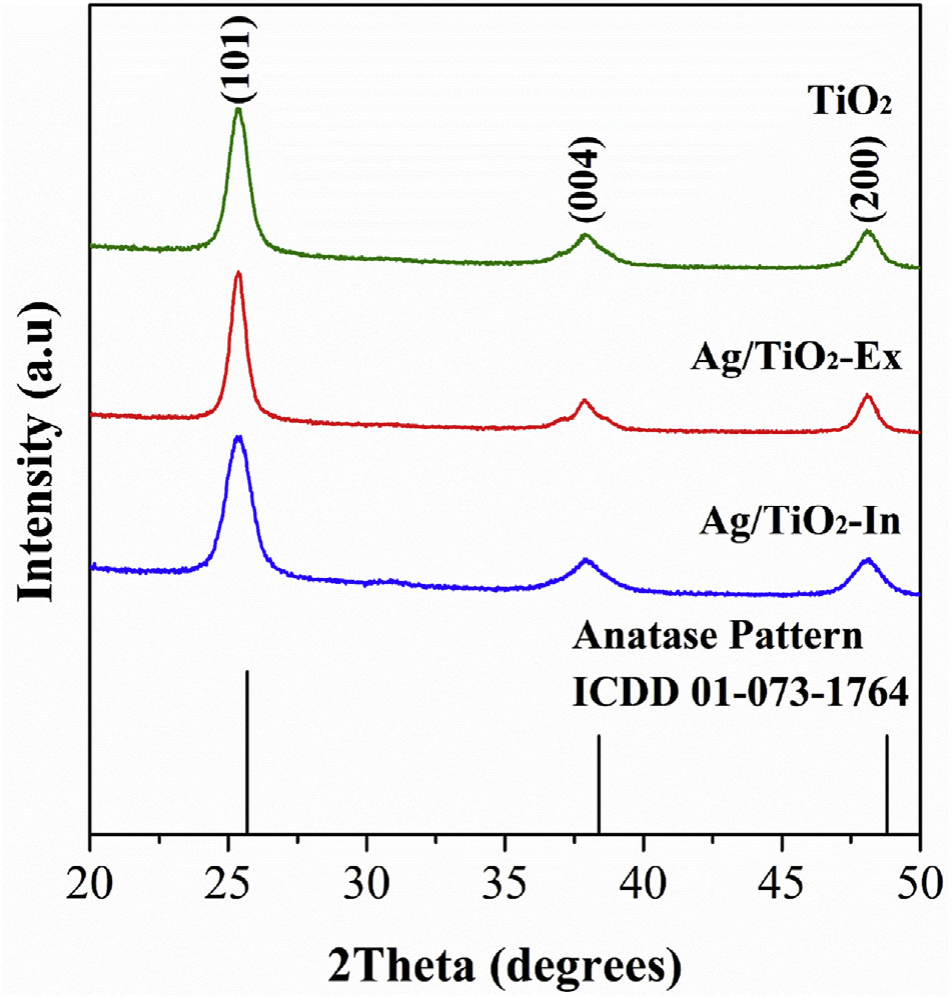
XRD pattern of TiO_2_ nanoparticles and nanoparticles of silver-modified TiO_2_ by *Ex situ* method (Ag/TiO_2_-Ex) and nanoparticles of silver-modified TiO_2_ by *In situ* method (Ag/TiO_2_-In).

Fig. 1 shows the X-ray diffraction pattern of synthesized and modified TiO_2_ nanoparticles. The analysis of the three materials (TiO_2_, Ag/TiO_2_-Ex and Ag/TiO_2_-In), showed anatase-phase TiO_2_ with characteristic diffraction peaks of 2θ values at about 25.3°, 37.8°, 48° which are attributed to the (101), (004), (200) planes, respectively. These results are consistent with the reported by [20, 21] and coincided with standard ICDD card anatase (01-073-1764). On other hand, in the XRD patterns of Ag/TiO_2_ materials, no crystalline structures associated with Ag or Ag_2_O were identified, this probably due to the low metal content (1% wt.) in samples or a high distribution of Ag nanoparticles.

**Fig. 2:**
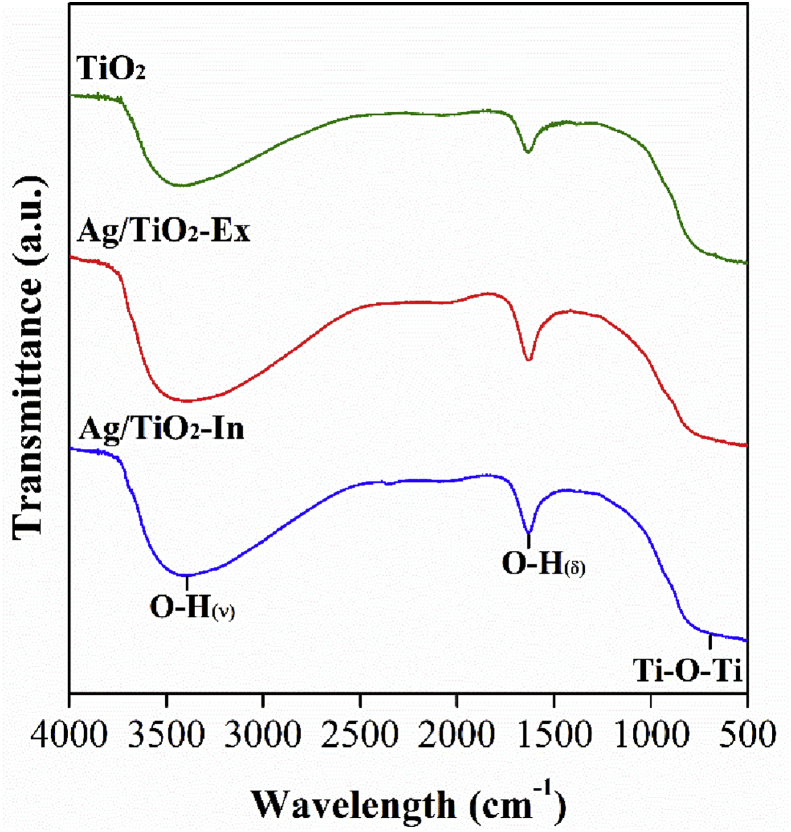
FTIR spectrum of TiO_2_ nanoparticles and nanoparticles of silver-modified TiO_2_ by *Ex situ* method (Ag/TiO_2_-Ex) and nanoparticles of silver-modified TiO_2_ by *In situ* method (Ag/TiO_2_-In).

The FTIR spectra obtained for pure and modified TiO_2_ are shown in Fig. 2. Both modified samples and raw TiO_2_ exhibited similar vibration patterns. Three main bands could be observed in all samples. The broad band observed between 3000 and 3377 cm^−1^ corresponds to stretching vibration of O-H bond (can be attributed to the chemisorption and/or physisorption to the water at the surface of the particles). The band centered at 1610 cm^−1^ is characteristic of bending vibration mode of O-H bond. The characteristic bands of titanium dioxide are located at 400 and 800 cm^−1^ and they are associated with the elongated Ti-O-Ti. However, according to Bagheri et al. [22], the vibrations associated with the metal support bonding were not observed in the spectra because these vibrations are in the far infrared region (below 400 cm^−1^) [23]. From these results, no larger modification in terms of functional groups at the particles surface was obtained.

**Fig. 3:**
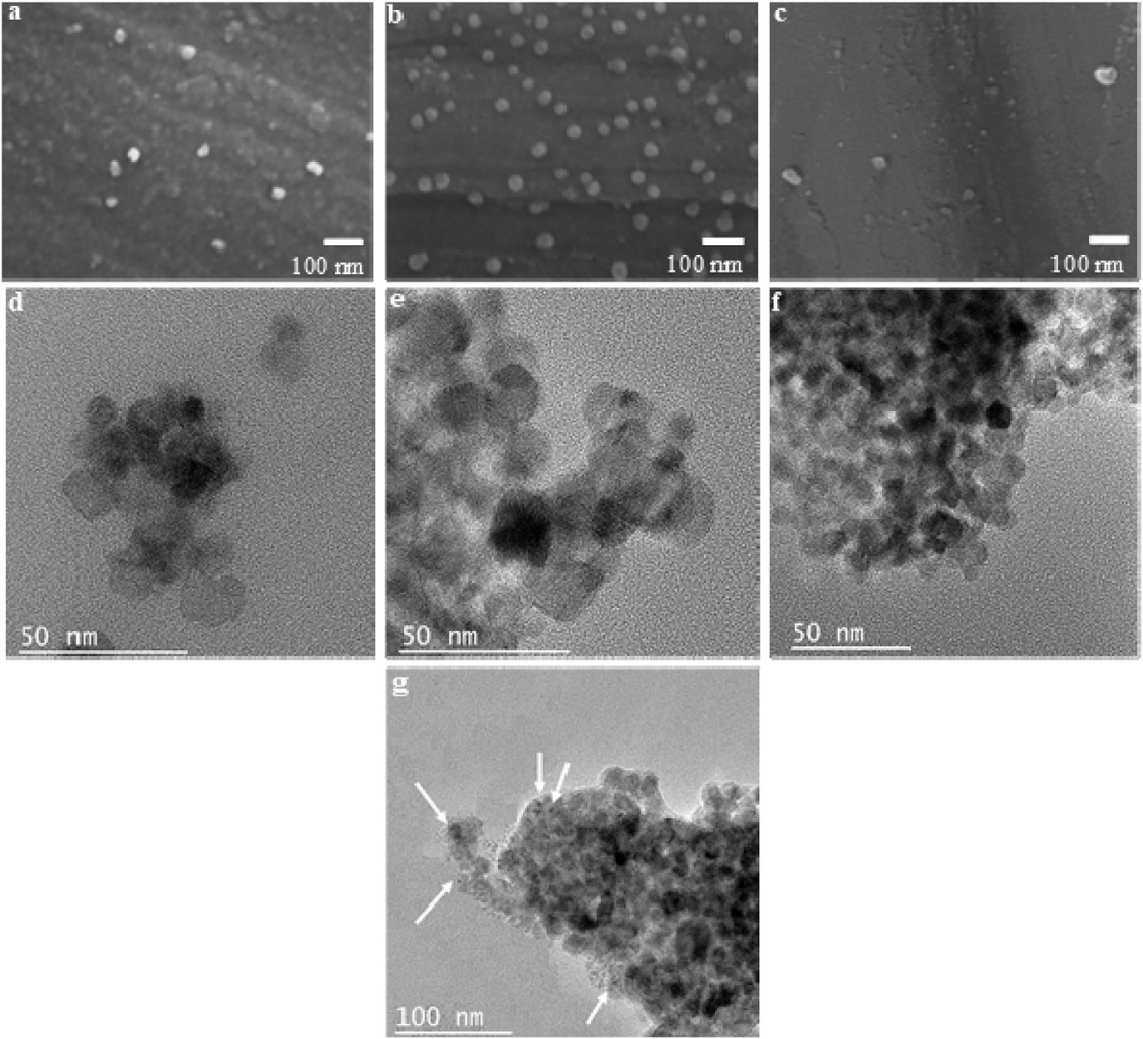
SEM micrographs. a) TiO_2_ nanoparticles b) Silver-modified TiO_2_ nanoparticles (E*x situ* method, Ag/TiO_2_-Ex) c) Silver-modified TiO_2_ nanoparticles (*In situ* method, Ag/TiO_2_-In). TEM micrographs d) TiO_2,_ nanoparticles e) Silver-modified TiO_2_ nanoparticles (E*x situ* method, Ag/TiO_2_-Ex) f) Silver-modified TiO_2_ nanoparticles (*In situ* method, Ag/TiO_2_-In) g) Ag decorated TiO_2_ nanoparticles by *Ex situ* method.

Fig. 3 shows SEM (Fig. 3. a–c) and TEM (Fig. 3. d–g) images of the TiO_2_ nanoparticles synthesized and silver modified TiO_2_ nanoparticles. In all cases a similar morphology can be observed for the TiO_2_ (Fig. 3a), Ag/TiO_2_-Ex (Fig. 3b) and Ag/TiO_2_-In (Fig. 3c) samples. The images reveal that nanoparticles have a spherical morphology without large agglomerates. TEM images (Fig 3 d–g, 145.000 kX) confirm the morphology observed by SEM analysis. No significant differences were observed at these magnifications. However, when the Ag/TiO_2_-Ex sample was observed at higher magnifications (Fig. 3g) some TiO_2_ particles decorated with Ag were found as pointed by the white arrows. Similar results have been previously reported by other authors [24, 25, 26].

**Fig. 4:**
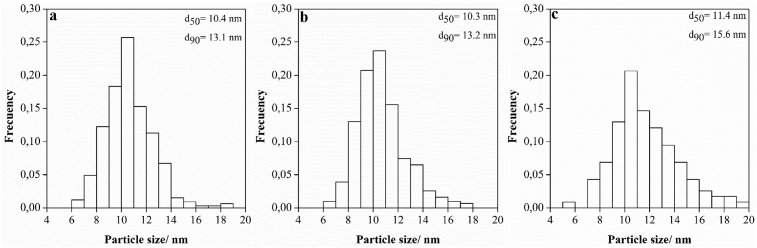
Histogram of TiO_2_ particle size distribution by TEM. a) TiO_2_ nanoparticles b) Silver-modified TiO_2_ nanoparticles (E*x situ* method, Ag/TiO_2_-Ex) c) Silver-modified TiO_2_ nanoparticles (*In situ* method, Ag/TiO_2_-In).

The average diameter of dispersed NPs was measured by the image J software and the results are shown in Fig. 4. The histograms of the particle size distribution and the corresponding d50 and d90 are included in the same figure. In all cases, a narrow particle size distribution was observed, with mean sizes 10.6 ± 1.9 nm, 10.7 ± 1.9 nm and 11.8 ± 2.6 nm for TiO_2_, Ag/TiO_2_-Ex, Ag/TiO_2_-In respectively. These results are in agreement with the observed from SEM and TEM images, where a slight increment in the mean particle size was achieved for the *In situ* method. This increment can be explained by the experimental procedure used to obtain TiO_2_ nanoparticles modified *In situ* with silver precursor. The modification of the hydrolysis speed and the condensation process could have favored the formation of greater particles. It is emphasized that particles in the nanoscale have a great surface area and smaller particle size can favor the photocatalytic activity of the material [27].

Chemical composition of pure and modified TiO_2_ was obtained by EDS through statistical analysis of at least four spectra. The Ag presence was observed in the spectra of both modification methods, this confirms that the two methods used for the modification of TiO_2_ nanoparticles with silver were successful. Weight percentage (wt%) of Ag was 1.20 ± 0.39 and 1.21 ± 0.32 for Ag/TiO_2_-Ex and Ag/TiO_2_-In respectively, values close to 1 wt% calculated theoretically. On the other hand, in the spectra the presence of Ti and O was also found. Moreover, the other peaks observed in the spectrum are attributed to gold used to coat the titanium dioxide and the support employed during the observation (Fig. S1, supplementary data).

**Fig. 5:**
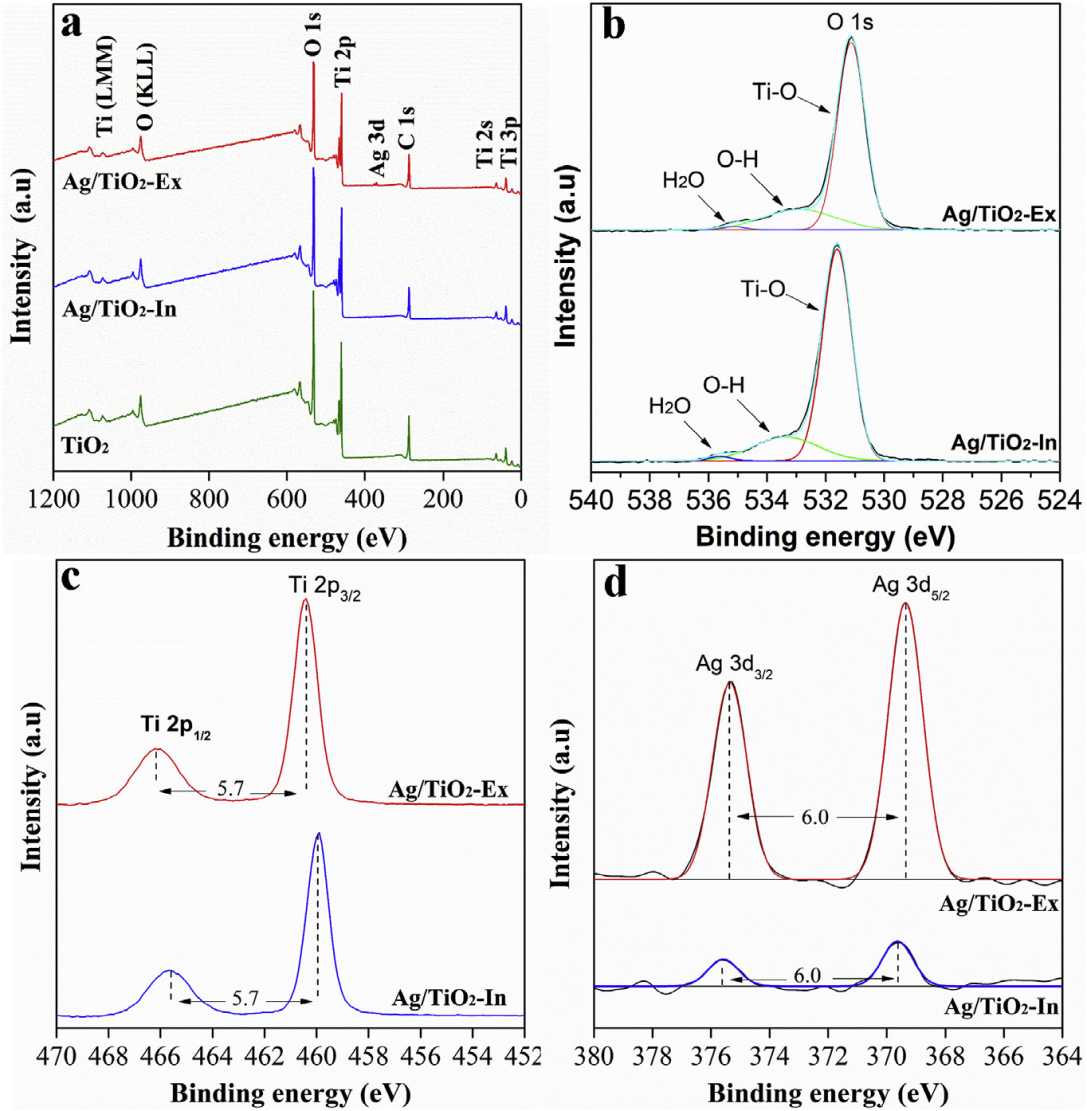
a) Survey spectra of TiO_2_ and Ag/TiO_2_ by both methods b) O 1s spectra for Ag/TiO_2_ c) Ti 2p spectra for Ag/TiO_2_, d) Ag 3d spectra for Ag/TiO_2_ samples.

Surface components and valence states in Ag/TiO_2_-Ex and Ag/TiO_2_-In composites were studied by XPS. High resolution XPS spectra of O 1s, Ti 2p and Ag 3d were obtained. Fig. 5a shows the survey spectra of TiO_2_ and Ag/TiO_2_ materials prepared by both methods. XPS peaks show merely peaks corresponding with Ti, O, Ag and a C. The last element is related to the adventitious hydrocarbon from XPS instrument itself. For Oxygen (Fig. 5b) Three peaks were identified: O 1s at the binding energies of 531.13, 532.89 eV and 535.1 eV for Ag/TiO_2_-Ex and 531.6, 533.35 and 535.5 for Ag/TiO_2_-In associate to O in TiO_2_, hydroxyl species and H_2_O (moisture) [28, 29], differences could be related with a lower concentration of Ti atoms in the Ag/TiO_2_- In sample that cause a displacement to higher binding energy values [30]. The peaks of Ti 2p _3/2_ and Ti 2p _1/2_ (Fig. 5c) were found at 459.9 and 465.6 eV for Ag/TiO_2_-In and 460.5 and 466.2 eV Ag/TiO_2_-Ex respectively and his bending splitting of 5.7 eV, indicating an oxidation state of Ti^4+^ in TiO_2_ [31]. It is important to point out that no trace of Ti^3+^ or Ti^2+^ was found. Fig. 5d shows a double peak with intensities of Ag 3d_5/2_ and Ag 3d_3/2_ centered at 369.4 and 375.4 eV for Ag/TiO_2_-Ex and 369.6 and 375.6 eV for the Ag/TiO_2_-In, respectively. The splitting of the Ag 3d doublet was 6.0 eV and this binding energy indicated that Ag mainly existed in the Ag^0^ state on the both materials [32, 33]. The decrease in the intensity of the peaks of Ag/TiO_2_-In, compared to the peaks of the sample Ag/TiO_2_-Ex, are related with that Ag is located at the surface of TiO_2_ in the *Ex situ* synthesis method. The shifting of the bands towards higher binding energy can be related to an decrease in particle size as reported in the literature and that is in agreement with TEM results [32, 33].

### 4.2 Bactericidal activity

**Fig. 6:**
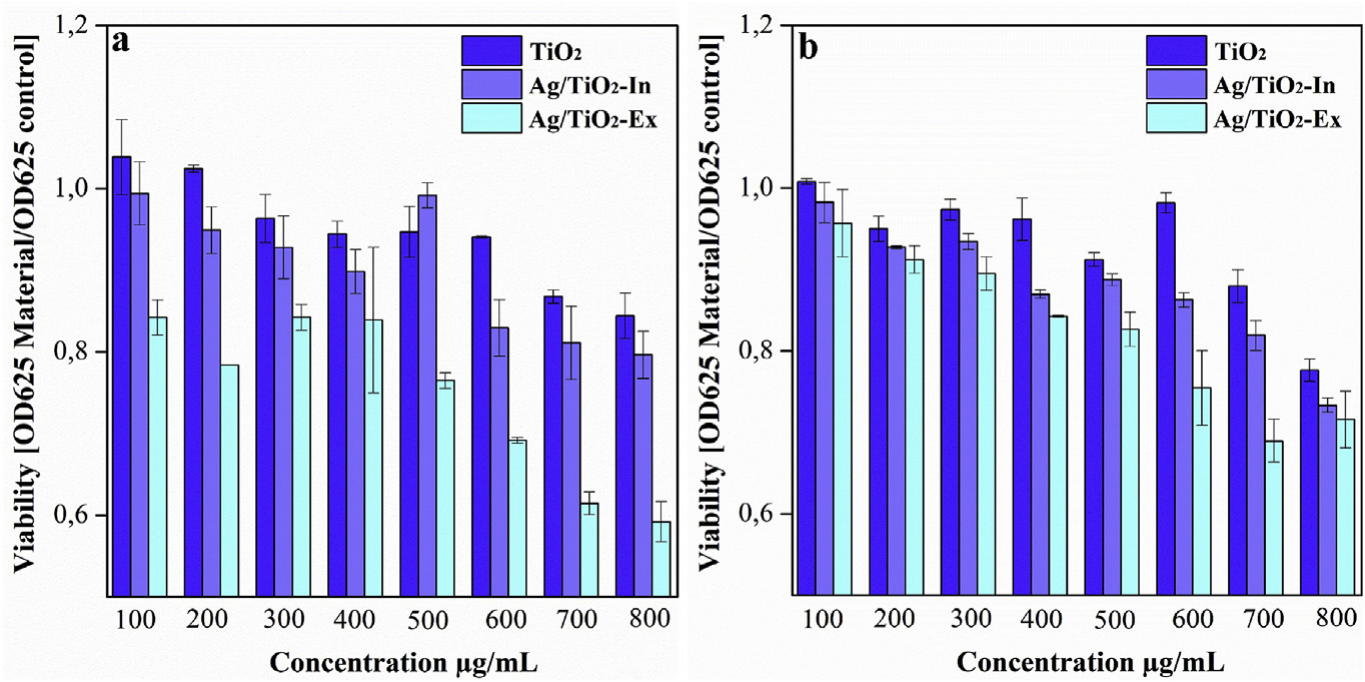
a) Viability of *E. coli* subjected to TiO_2,_ Ag/TiO_2_-Ex and Ag/TiO_2_-In b) Viability of *S. aureus* subjected to TiO_2,_ Ag/TiO_2_-Ex and Ag/TiO_2_-In.

Fig. 6a shows the bacterial viability of *E. coli* under different concentrations of TiO_2_, Ag/TiO_2_-Ex and Ag/TiO_2_-In. By the broth macrodilution method, the bactericidal properties of TiO_2_, Ag/TiO_2_-Ex and Ag/TiO_2_-In were evaluated. As expected, bacterial viability decreased in a concentration-dependent manner. However, an improvement is noted in the materials containing Ag, related to the bactericidal properties of this metal [34](TiO_2_ vs. Ag/TiO_2_-In: p = 0.0048; TiO_2_ vs. Ag/TiO_2_-Ex: p = 0.000038e-6). It was also noted an improvement of bacterial activity of the Ag/TiO_2_-Ex modification compared with the Ag/TiO_2_-In modification (p = 0.00022e-3), associated with an increased contact with the medium, since Ag is located at the surface of TiO_2_ by the synthesis method. The TiO_2_ particles modified with Ag were tested in a Streptococcus strain. It was found that the Ag modification also enhanced antimicrobial activity of TiO2 particles under that bacteria (TiO_2_ vs. Ag/TiO_2_-Ex: p=0.000001; TiO_2_ vs. Ag/TiO_2_-In: p=0.0013). Consequently, the viability of bacteria exposed to the modification using the *Ex situ* method was observed. A significant reduction of the viability of bacteria subjected to particles modified with Ag via *In situ* (Ag/TiO_2_-Ex vs. Ag/TiO_2_-In: *p* = 0.0084) was found (see Fig. 6b). The bactericidal activity of the tested materials was effective for both strains of bacteria. In addition, the *Ex situ* modification method showed better results in both cases.

MIC50 is defined as the minimum amount of a drug/compound to inhibit 50% of a microorganism growth. Prediction values of MIC50 for *E. Coli* and *S. aureus* exposed to with TiO_2_ and TiO_2_ modified with silver by both methods is shown in Table 1. The decrease in the concentration of the material in the samples Ag/TiO_2_-Ex and Ag/TiO_2_-In when it is compared to TiO_2_ agrees with the results reported by Vallejo et al. [35]. Ag/TiO_2_-Ex requires a predicted lower concentration to reach the MIC50 than Ag/TiO_2_-In, therefore; resulting in a higher bactericidal activity. This result suggests that the modification of TiO_2_ particles with Ag using an *Ex situ* method improves the antimicrobial activity of TiO_2_, which agrees with the viability values after material exposition (Fig. S2 and S3, Supplementary data). The variations in the results of bactericidal activity and MIC50 prediction, are related with the antimicrobial test (broth macrodilution) implemented in this study.

**Table 1:** MIC50 prediction values for pure and modified TiO_2_ against *E. coli* and *S. aureus* bacteria.

Material	*E. coli*	*S. aureus*
TiO_2_	1735,4 μg/mL	1294 μg/mL
Ag/TiO_2_-Ex	1263,7 μg/mL	964,3 μg/mL
Ag/TiO_2_-In	1459,1 μg/mL	1078,6 μg/mL

## 5 Conclusions

TiO_2_ nanoparticles were obtained by hydrothermal method and modified TiO_2_ nanoparticles with silver were obtained by wet impregnation (*Ex situ*) and *In situ*. The mean size of the particles was controlled by manipulating the speed of the hydrolysis and condensation reaction. For TiO_2_ and Ag/TiO_2_-Ex the size was ∼10.6 nm and for the Ag/TiO_2_-In was ∼11.8 nm. The anatase crystalline phase was obtained controlling the calcination temperature. The temperature did not exceed 600 °C to avoid a crystalline phase change.

The results obtained from bactericidal activity showed that the addition of Ag using two synthesis resulted in an improvement of antibacterial activity compared with unmodified TiO_2_. Moreover, activity of TiO_2_ nanoparticles with silver addition was increased by *Ex situ* method when compared to TiO_2_ and Ag/TiO_2_-In.

## Declarations

### Author contribution statement

G. Durango-Giraldo, A. Cardona, Juan F. Zapata: Conceived and designed the experiments; Contributed reagents, materials, analysis tools or data; Wrote the paper.

Juna F. Santa, R. Buitrago-Sierra: Performed the experiments; Analyzed and interpreted the data; Wrote the paper.

### Funding statement

This research did not receive any specific grant from funding agencies in the public, commercial, or not-for-profit sectors.

### Competing interest statement

The authors declare no conflict of interest.

### Additional information

No additional information is available for this paper.

## Appendix A Supplementary data

Supplementary data_V3 Supplementary data_V3
