# Supplementary material for: Titanium dioxide modified with silver by two methods for bactericidal applications
Source: Heliyon. 2019 May 14;5(5):e01608. doi: 10.1016/j.heliyon.2019.e01608 (PMC6522664; doi:10.1016/j.heliyon.2019.e01608)
Supplement: Supplementary data_V3 [file mmc1.pdf]

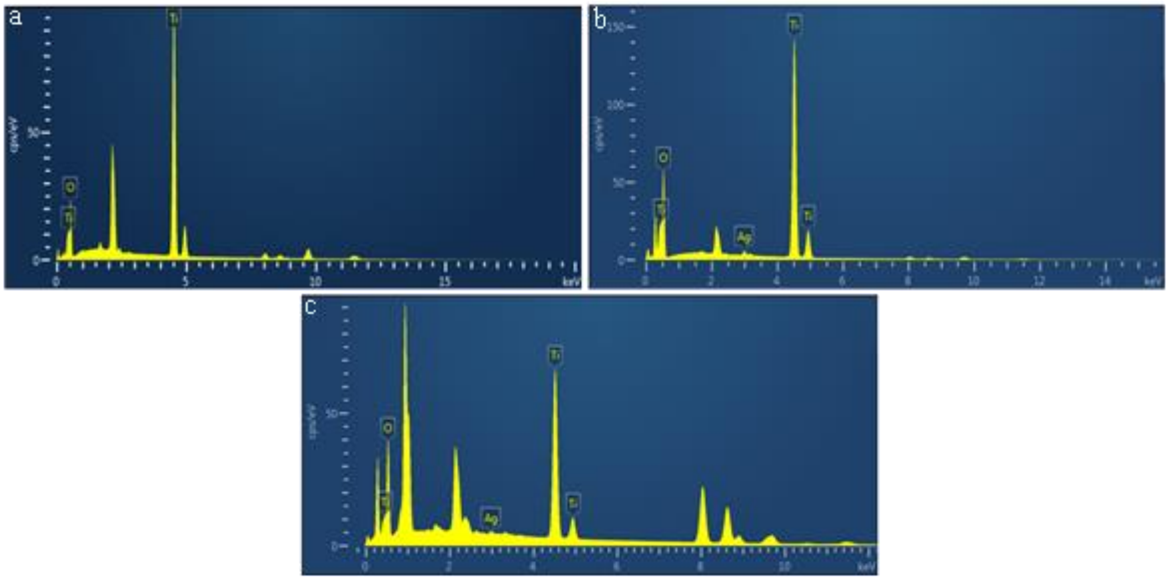

Figure S1: EDS spectra a) TiO_2 nanoparticles. b) nanoparticles of silver-modified TiO_2 by *Ex situ* method (Ag/TiO_2-Ex) c) nanoparticles of silver-modified TiO_2 by method (Ag/TiO_2- In).

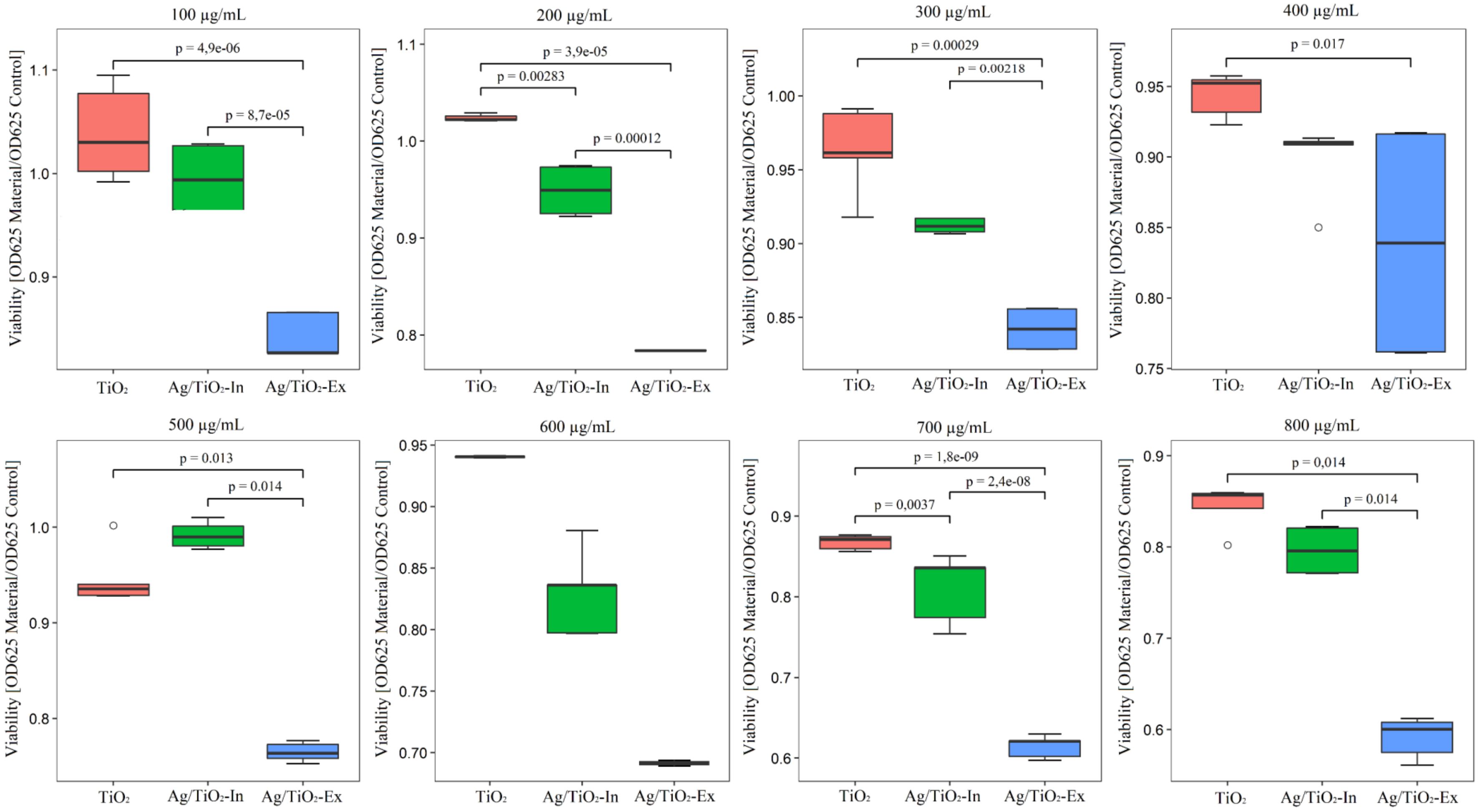

Figure S2. Box plots for *E.coli* viability subjected to TiO_2, Ag/TiO_2-In and Ag/ TiO_2-Ex at different concentration

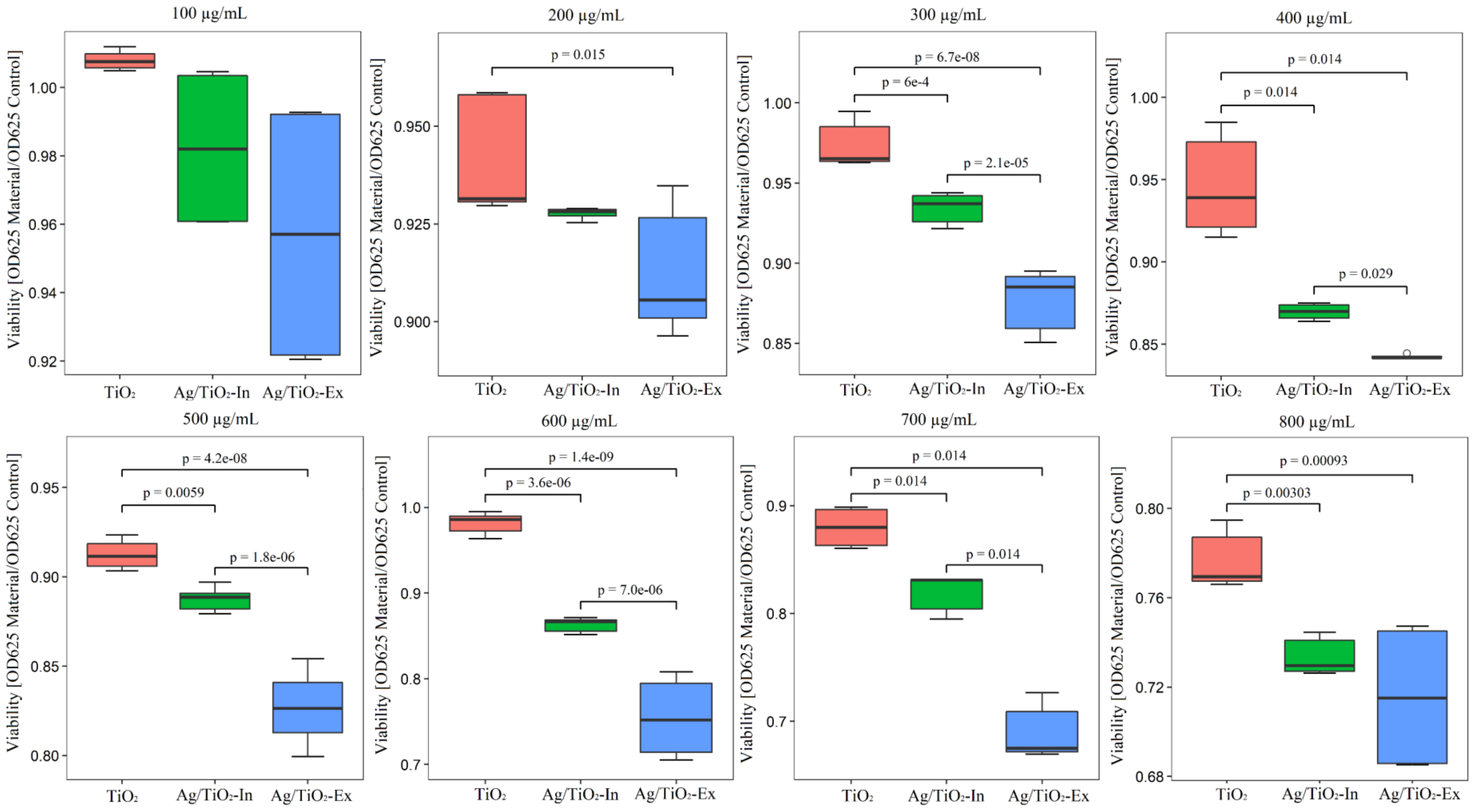

Figure S3. Box plots for *S.aureus* viability subjected to TiO_2, Ag/TiO_2-In and Ag/TiO_2-In Ag/ TiO_2-Ex at different concentration
